# Collagen–ORC Versus Standard Treatment in Diabetic Foot Ulcers: A Systematic Review and Meta‐Analysis of Randomised Trials

**DOI:** 10.1111/iwj.70782

**Published:** 2025-11-17

**Authors:** George Theodorakopoulos, David G. Armstrong

**Affiliations:** ^1^ Wound and Foot Specialist Athens Greece; ^2^ Technological Educational Institute of Patras Patras Greece; ^3^ Queen Margaret University Edinburgh UK; ^4^ University of West Attica Aigaleo Greece; ^5^ University of Thessaly Larisa Greece; ^6^ National and Kapodistrian University of Athens Athens Greece; ^7^ Southwestern Academic Limb Salvage Alliance (SALSA) Los Angeles California USA; ^8^ Center to Stream Healthcare in Place (C2SHiP) Alexandria Virginia USA; ^9^ Keck School of Medicine University of Southern California Los Angeles California USA

**Keywords:** collagen dressing, collagen–oxidised regenerated cellulose (collagen–ORC), diabetic foot ulcers, meta‐analysis, PRISMA, standard of care, wound healing

## Abstract

Diabetic foot ulcers (DFUs) are a major cause of infection, hospitalisation, and amputation. Collagen‐based dressings—especially collagen combined with oxidised regenerated cellulose (ORC)—are proposed to improve healing by modulating matrix metalloproteinases (MMPs), stabilising the extracellular matrix (ECM), and tempering inflammation; some formulations also include antimicrobial or bioactive adjuncts. We conducted a systematic review of randomised controlled trials (RCTs) following Preferred Reporting Items for Systematic Reviews and Meta‐Analyses (PRISMA) guidance. Adults with DFUs were eligible. Interventions included collagen‐alone or collagen‐combination dressings (e.g., collagen–oxidised regenerated cellulose [collagen–ORC]/silver, collagen–chitosan) versus standard of care (SOC) or alternative dressings. To ensure comparable outcomes, the quantitative synthesis was pre‐specified and restricted to complete wound closure (yes/no, intention‐to‐treat [ITT]) from collagen‐combination RCTs with aligned constructs; other outcomes were synthesised narratively. Meta‐analyses were performed in Microsoft Excel using Mantel–Haenszel methods for risk ratios (RR) with a fixed‐effect primary model and DerSimonian–Laird random‐effects sensitivity analysis; heterogeneity was summarised with Cochran's Q, between‐study variance (*τ* ^2^), and Higgins' *I* ^2^ statistic (*I* ^2^), and a 95% prediction interval was reported for random‐effects. (Protocol not registered). Six studies (five randomized controlled trials and one single‐blinded non‐randomized comparative study; total *n* = 314) met inclusion. In a focused meta‐analysis of the two collagen‐combination RCTs, treatment was associated with a higher probability of complete wound closure versus control (RR 1.69, 95% confidence interval [CI] 1.05–2.72; *I* ^2^ = 0%). One assessor‐blinded RCT of collagen alone reported higher 12‐week closure versus a placebo dressing and was not pooled due to heterogeneity. Across studies, signals also favored collagen‐based care for earlier area reduction and, in one trial, fewer infection‐related withdrawals; mechanistic work showed reductions in MMP‐9/TIMP‐2. However, most trials were small and single‐centre, comparators and adjuncts varied, follow‐up was short (~8 days–24 weeks, clinical endpoints typically 4–20 weeks), outcome definitions were non‐standardised, and key confounders (off‐loading, infection management, vascular status, glycaemic control) were inconsistently addressed. Collagen‐based dressings—particularly collagen‐combination formulations—appear to improve complete closure when added to the standard of care (SOC) for diabetic foot ulcers (DFUs), but the evidence is limited by study size, heterogeneity, and risk of bias. Larger, prospectively registered, multicentre RCTs with standardised outcomes and longer follow‐up are needed to define clinical and cost‐effectiveness and to identify which patients benefit most. Collagen–ORC dressings show promise as an adjunctive treatment for DFUs by influencing the inflammatory microenvironment and supporting tissue repair. However, the certainty of the current evidence remains limited, highlighting the need for further high‐quality randomised studies.

**Summary**

- In randomized trials, collagen‐ORC and other collagen‐combination dressings used adjunctively to standard care were associated with a higher chance of complete closure versus comparators.
- Individual studies also showed earlier wound‐size reduction and protease‐modulating effects.
- Certainty remains limited by small, mostly signle‐center RCTs, heterogeneous comparators/endpoints, and short follow up‐larger, trials are needed.

**Abbreviations**
ANOVA: analysis of variance
AutoCAD: AutoCAD (computer‐aided design software by Autodesk)
AWD: advanced wound dressing
CASP: Critical Appraisal Skills Programme
CI: confidence interval
Cochran's *Q*
: test statistic for heterogeneity across studies
CSWD: conservative sharp wound debridement
CT: computed tomography
DFU: diabetic foot ulcer
DPN: diabetic peripheral neuropathy
ECM: extracellular matrix
effect size: summary measure of treatment effect (e.g., RR, OR, MD, SMD)
Egger's test: regression test for funnel‐plot asymmetry
eKare: eKare InSight Wound Measurement System
ELISA: enzyme‐linked immunosorbent assay
FT3: free triiodothyronine
FT4: free thyroxine
GRADE: Grading of Recommendations, Assessment, Development and Evaluation
H^2^
: heterogeneity index (total/within‐study variance ratio)
Hartung–Knapp–Sidik–Jonkman (HKSJ): random‐effects CI/*p*‐value adjustment
*I* ^2^
: percent of variability due to heterogeneity (not chance)
IL‐1β: interleukin‐1 beta
ITT: intention‐to‐treat
Kaplan–Meier: Kaplan–Meier survival analysis
LOPS: loss of protective sensation
MD: mean difference
MeSH: medical subject headings
MMP: matrix metalloproteinase
MMP‐1: matrix metalloproteinase‐1 (collagenase‐1)
MMP‐8: matrix metalloproteinase‐8 (neutrophil collagenase)
MMP‐9: matrix metalloproteinase‐9 (gelatinase B)
OR: odds ratio
ORC: oxidised regenerated cellulose
PAD: peripheral arterial disease
PICO: population, intervention, comparator, outcome
PMVT: processed microvascular tissue
PRISMA: Preferred Reporting Items for Systematic Reviews and Meta‐Analyses
QoL: quality of life
RCT: randomised controlled trial
REML/ML: restricted/maximum likelihood estimators for *τ* ^2^
RR: risk ratio (relative risk)
RT‐PCR: reverse transcription polymerase chain reaction
Sidik–Jonkman (SJ): τ^2^ estimator; foundation for HKSJ adjustment
SMD (Hedges g): standardised mean difference
SOC: standard of care
SoF: summary of findings (table)
TCC: total contact cast
TIMP: tissue inhibitor of metalloproteinases
TIMP‐1: tissue inhibitor of metalloproteinases‐1
TIMP‐2: tissue inhibitor of metalloproteinases‐2
Visitrak: Visitrak Digital Planimetry System
*τ* ^2^
: between‐study variance (random‐effects)

## 1 Introduction

### 1.1 Global and Clinical Relevance of Diabetes Mellitus

Diabetic foot ulcers (DFUs) are a frequent and serious complication of diabetes mellitus, affecting up to 34% of patients during their lifetime [1]. Approximately 85% of non‐traumatic lower‐limb amputations are preceded by DFUs [2]. Mortality remains high, with estimated rates of 5% at 1 year and 42% at 5 years after ulcer onset [2]. Beyond these outcomes, DFUs substantially increase morbidity, reduce quality of life (QoL), and drive greater utilisation of healthcare services [3].

### 1.2 Socioeconomic Consequences of Diabetic Foot Ulcers

Chronic wounds, including DFUs, affect approximately 2.5% of the US population and place a substantial economic burden on healthcare systems [4]. With population aging, the global rise in diabetes and obesity, and persistent challenges with infection, DFUs are expected to remain a major clinical, social, and economic issue. Their management is resource‐intensive, often requiring frequent outpatient visits, hospitalisation, surgical intervention, prolonged antibiotic use, and specialised wound care [1]. In the United States, annual DFU‐related costs exceed $100 billion, with average annual expenditure about $58 000 per patient compared with $17 000 in ulcer‐free individuals with diabetes [5]. In Europe, the mean cost per patient is €10 931, rising sharply in the event of amputation [6]. Beyond financial impact, DFUs reduce mobility, contribute to social isolation, and frequently lead to depression [7].

### 1.3 Pathophysiology of Diabetic Foot Ulcers

**FIGURE 1:**
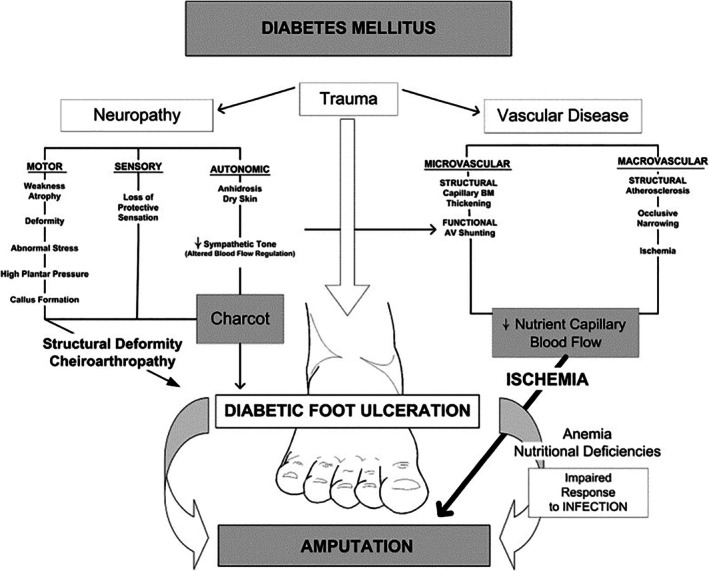
Pathophysiology of diabetic foot ulcers [10].

DFUs typically arise from repetitive mechanical trauma—either microtrauma from daily ambulation or macrotrauma from acute injury—acting on an insensate foot. This risk is amplified by diabetic peripheral neuropathy (DPN), which causes loss of protective sensation (LOPS) and leaves patients unaware of minor injuries [1, 8]. Biomechanical deformities such as hammertoes, plantarflexed metatarsals, equinus, and Charcot arthropathy concentrate abnormal plantar pressures, further increasing the risk of breakdown [9, 10]. Peripheral arterial disease (PAD), though often overlapping with diabetes, represents a distinct pathology [11, 12]. While PAD rarely initiates ulceration directly, it reduces blood flow, promotes ischaemia and tissue hypoxia, and delays healing. PAD is present in up to 50% of DFUs and, in combination with neuropathy, is the strongest predictor of ulcer onset, chronicity, and poor outcomes (Figure 1) [9, 10, 11, 12].

### 1.4 Infection, Biofilm, and Clinical Management in DFU Healing

Infection and biofilm are major barriers to DFU healing. Biofilms are present in 60%–100% of chronic DFUs [13]. These structured microbial communities, commonly including *Staphylococcus aureus* and *Pseudomonas aeruginosa* , protect pathogens from host immune responses and antibiotics, driving chronic inflammation, delayed granulation, and higher amputation risk [14]. Effective management requires a comprehensive approach targeting local and systemic factors. Locally, conservative sharp wound debridement (CSWD) [14] and topical antiseptics are essential for biofilm disruption. Systemically, timely empirical antimicrobial therapy guided by clinical indicators is necessary, as chronic infections substantially increase amputation risk [15]. Beyond infection control, successful DFU healing requires multifaceted interventions: (a) strict glycaemic control to reduce inflammation, oxidative stress, and impaired immunity [16], (b) surgical debridement to remove necrotic tissue and reduce bacterial load [1], (c) off‐loading—with the total contact cast (TCC) considered the gold standard [17], and (d) maintenance of a moist wound environment with advanced wound dressings (AWDs). Traditional wet‐to‐dry dressings are now considered obsolete and are not recommended due to pain, trauma to healing tissue, and higher infection risk. While AWDs protect and promote granulation, they often fail to adequately regulate the inflammatory phase [9, 10, 15, 16, 18].

### 1.5 Multifactorial Barriers to DFU Healing

Beyond DPN and vascular disease, patients frequently exhibit metabolic abnormalities that worsen healing and disease severity. These include low albumin; low thyroid hormones—free triiodothyronine (FT3) and free thyroxine (FT4); low iron and bilirubin—markers of poor nutrition, slowed metabolism, and low antioxidant capacity. Elevated uric acid, lipoprotein(a), and triglycerides, along with impaired kidney function are also common, contributing to ischaemia and inflammation. These factors strongly correlate with more severe vascular lesions on computed tomography (CT) angiography, suggesting the metabolic profile plays a key role in DFU progression [19].

### 1.6 Cellular and Molecular Basis of DFU Non‐Healing

**FIGURE 2:**
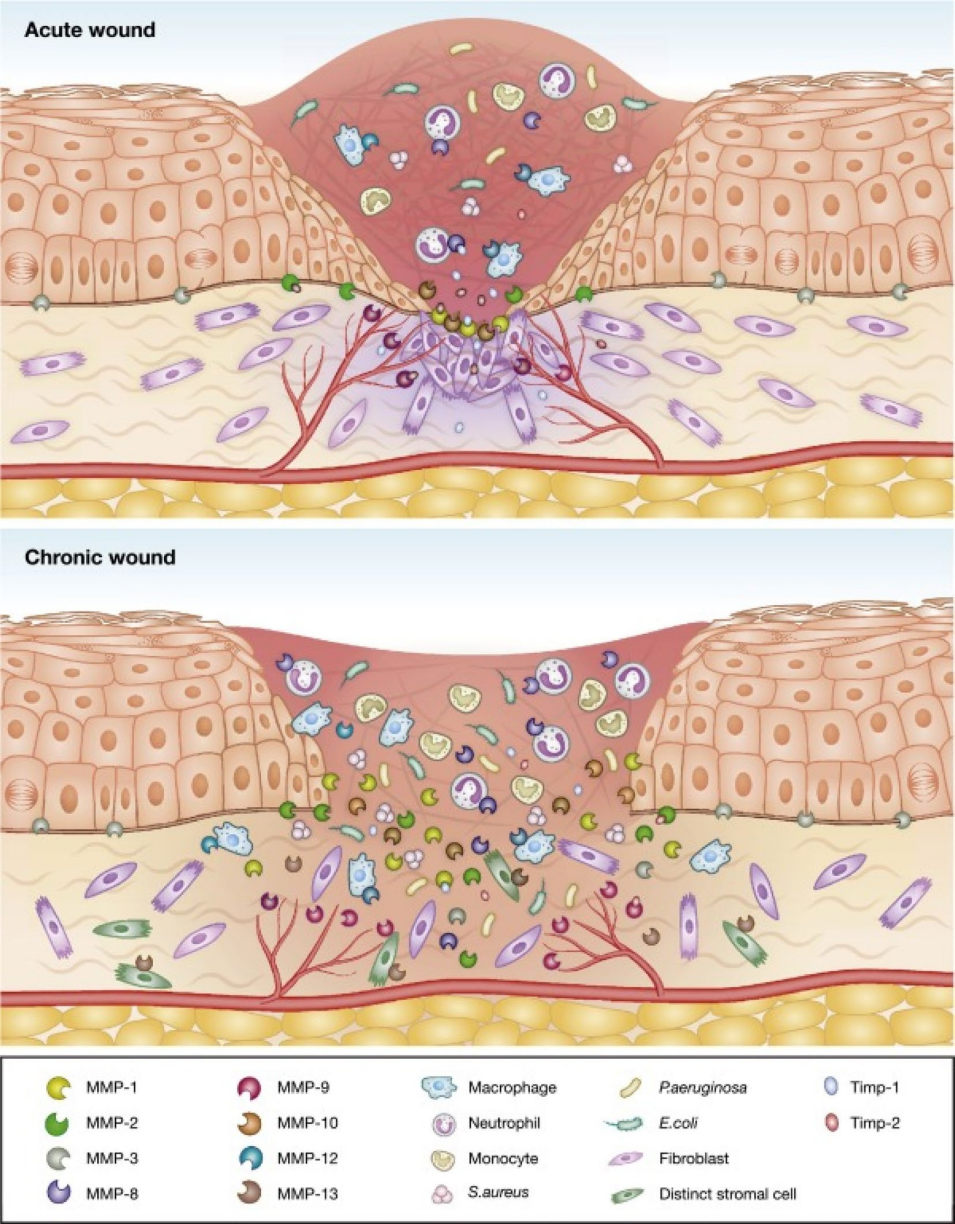
Representation of acute and chronic wound states with dysregulated matrix metalloproteinases (MMPs) [24].

Chronic DFUs are characterised by a prolonged inflammatory phase with excess pro‐inflammatory cytokines and matrix metalloproteinases (MMPs)—notably MMP‐1, MMP‐8, and MMP‐9—leading to excessive extracellular matrix (ECM) degradation and impaired repair [20]. MMP‐1 (collagenase‐1) facilitates keratinocyte migration and skin remodelling, but its activity must be balanced by tissue inhibitor of metalloproteinases‐1 (TIMP‐1) to prevent tissue damage [21]. Muller et al. [21] showed that the MMP‐1/TIMP‐1 ratio is prognostic; a ratio ≥ 0.39 was associated with favourable healing. In well‐healing ulcers, MMP‐8 and MMP‐9 levels drop early, while MMP‐1 rises by week two, highlighting the importance of balanced protease activity. In addition, MMP‐2 and MMP‐9 contribute to DFU chronicity by degrading ECM, destroying growth factors, and impairing angiogenesis [22]. Lobmann et al. reported that collagen–oxidised regenerated cellulose (ORC) dressings reduced the MMP‐9/TIMP‐2 ratio alongside improved clinical healing signals [22]. Consistently, Schultz and Wysocki emphasised that while controlled protease activity is essential for normal healing, chronically elevated MMPs perpetuate ECM degradation and impair wound closure [23]. These data underscore the need to target multiple MMP pathways rather than focusing solely on MMP‐1/TIMP‐1 dynamics (Figure 2).

### 1.7 Rationale and Objectives

Because persistent inflammation and protease imbalance are central to DFU chronicity, bioactive dressings that modulate the wound microenvironment are biologically attractive. Collagen‐based technologies—particularly combinations with ORC or other adjuncts—aim to bind excess proteases, protect endogenous growth factors, and support granulation and re‐epithelialisation. This review undertakes a systematic synthesis of randomised controlled trials (RCTs) of collagen‐based dressings in DFUs and a focused meta‐analysis restricted to trials with comparable definitions of complete wound closure (yes/no, intention‐to‐treat [ITT]). Heterogeneous outcomes (e.g., early ‘responder’ definitions, biomarker surrogates) are synthesised narratively to preserve clinical validity. Mechanistically distinct comparators (e.g., processed microvascular tissue allograft) are included for context but not pooled with collagen interventions [25].

### 1.8 Bioactive Collagen Dressings in DFU Care

Collagen‐based dressings provide active support for wound repair by modulating excessive inflammation and promoting tissue regeneration. They help reduce MMP activity, preserve ECM, and stimulate angiogenesis and granulation; some formulations also demonstrate antimicrobial properties that limit pathogen proliferation. These mechanisms address key shortcomings of conventional AWDs, which provide moisture balance but may not adequately regulate the inflammatory phase. This review evaluates RCT evidence on collagen‐based dressings for DFUs, with specific emphasis on collagen–ORC formulations. While the focus is on ORC, collagen‐only and other combination dressings are included for completeness; results are presented separately by intervention type, and the quantitative synthesis is restricted to clinically comparable collagen‐combination trials reporting identical closure constructs.

## 2 Methods

### 2.1 Study Eligibility Criteria

To maximize rigor, we defined primary evidence set of RCTs for quantitative synthesis and allowed secondary mechanistic evidence set (non‐randomized) for narrative context only.

#### 2.1.1 Inclusion Criteria

Studies were eligible if they:

- Enrolled adults (≥ 18 years) with diabetic foot ulcers (DFUs) attributable to diabetic peripheral neuropathy (DPN), peripheral arterial disease (PAD), or both.
- Evaluated collagen‐based dressings: collagen alone, collagen–oxidised regenerated cellulose (collagen–ORC), or collagen combined with adjunctive components (e.g., silver, chitosan, growth factors).
- Were published in English between 2005 and 2025, with full‐text access and DFU healing reported as an outcome (clinical closure or validated wound endpoints).

**Evidence sets**

- Primary set (pre‐specified for pooling): Randomized controlled trials (RCTs).
- Secondary set (narrative only): Mechanistic/non‐randomized comparative studies. These studies were not combined in meta‐analysis.

#### 2.1.2 Exclusion Criteria

Studies were excluded if they were:

- Studies without a comparator; uncontrolled designs.
- Purely observational, animal, or in‐vitro studies.
- Review articles, meta‐analyses, conference abstracts, or non‐English publications.

**Notes on special cases**

- Mechanistically distinct RCT comparator: One RCT evaluated PMVT allograft vs collagen‐alginate. Because PMVT acts primarily via angiogenesis/perfusion (rather than protease modulation), it was retained for contextual comparison but not pooled with collagen‐based interventions.
- Single‐blinded non‐randomized study: Lobmann 2006 (biochemical emphasis over 8 days) was kept in the secondary set and excluded from all pooled RCT analyses.

### 2.2 Search Question Formulation Using PICO [ 26]

The review followed the Population, Intervention, Comparison, Outcome (PICO) framework to structure the search and research question:

- Population (P): Patients with diabetic foot ulcers.
- Intervention (I): Collagen‐based dressings (collagen alone, collagen–ORC, or collagen with adjuncts).
- Comparison (C): Standard of care (SOC) or advanced wound dressings (AWDs).
- Outcome (O): Improved or accelerated DFU healing.

Search terms were generated using the PubMed PICO Search Tool, combining MeSH terms with Boolean operators, for example, ‘diabetic foot ulcers’ AND ‘standard of care’ AND (‘collagen dressing’ OR ‘oxidized regenerated cellulose’ OR ‘collagen matrix’) AND ‘wound healing’.

#### 2.2.1 Literature Search and Study Identification

A structured search was conducted throughout March 2025, using four major biomedical indexing databases: PubMed, ScienceDirect, Scopus, and the Cochrane Library. These databases were chosen for their comprehensive coverage of peer‐reviewed literature. The search targeted RCTs evaluating collagen‐based dressings in managing stage I–II DFUs (Wagner or Texas 2A classification) [27, 28].

#### 2.2.2 Search Strategies by Database

- PubMed: 48 records identified → 17 screened → 2 included (1 duplicate removed).
- ScienceDirect: 4826 records identified → filtered and screened → 3 included.
- Scopus: 338 articles identified → 126 after filters → 1 included.
- Cochrane Library: 29 trials → 25 after filters → no new inclusions (duplicates already captured).

Each database used tailored Boolean combinations reflecting the PICO [26] components. Filters: randomised controlled trials, English language, and publication years 2005–2025.

### 2.3 Summary

In total, five primary RCTs and one comparative study met eligibility criteria. All were critically appraised using the Critical Appraisal Skills Programme (CASP) checklist [29]. The search strategy was transparent and methodologically sound, forming a solid basis for the review's findings. As noted above, one RCT evaluated PMVT versus collagen–alginate and is included for context but not pooled with collagen trials due to mechanistic differences [25]. Across included trials, potentially important confounders—systemic antibiotics, pressure off‐loading modality/adherence, vascular status, and comorbidities—were not consistently reported or controlled, which should be considered when interpreting results.

Meta‐analysis transparency note: The quantitative synthesis was pre‐specified for complete closure (yes/no, ITT) with a fixed‐effect primary model, DerSimonian–Laird random‐effects sensitivity, and full heterogeneity reporting (*Q* with *p* value, *I* ^2^, *τ* ^2^) plus a 95% prediction interval. All calculations and outputs are provided in Supplement S5 and the Excel workbook (DFU_meta_2025.xlsx).

### 2.4 Quantitative Synthesis Plan (Pre‐Specified, Focused)

To preserve clinical comparability and avoid inappropriate pooling:

#### 2.4.1 Primary Pooled Outcome (Meta‐Analysis)

Complete wound closure (yes/no, intention‐to‐treat [ITT]) at each trial's final prespecified follow‐up for collagen‐combination dressings vs. control (e.g., collagen–chitosan; collagen–ORC/silver).

- Effect measure: Risk ratio (RR).
- Model: Mantel–Haenszel fixed‐effect (primary) with DerSimonian–Laird random‐effects as sensitivity.
- Interpretation: Given *k* = 2, we place greater interpretive weight on the random‐effects interval and present both models transparently.
- Heterogeneity: Cochran's *Q* (with *p* value), between‐study variance (*τ* ^2^), and Higgins' *I* ^2^ (interpreted cautiously for *k* = 2).
- Random‐effects uncertainty: 95% prediction interval reported.

#### 2.4.2 Statistical Software and Reproducibility

Meta‐analyses were performed in Microsoft Excel (Microsoft Excel LTSC MSO Version 2408). The calculation workbook (DFU_meta_2025.xlsx) is provided in the Supplement with visible steps (2 × 2 inputs; study‐level log(RR) and SE; inverse‐variance weights; pooled fixed and random estimates; *Q*, *p*, *I* ^2^, *τ* ^2^; and 95% prediction interval). Continuity corrections were not required (no zero‐cell trials). All numerical results and tables are mirrored in Supplement S5.

#### 2.4.3 Non‐Pooled Outcomes (Narrative Synthesis)

Collagen‐alone (e.g., Park et al. (2019) 12‐week closure), early responder outcomes (e.g., ≥ 50% area reduction at 4 weeks), time‐to‐closure (hazard ratios), and biomarker endpoints (e.g., MMP‐9/TIMP‐2) were not pooled due to endpoint/timepoint or intervention heterogeneity [30].

#### 2.4.4 Unit‐of‐Analysis

One record per participant; if a trial reported multiple analysis sets/timepoints, we used the final prespecified follow‐up for the closure meta‐analysis and labelled any alternative endpoints narratively.

#### 2.4.5 Subgroup/Restricted Pooling Rationale

To ensure clinical and methodological comparability we pooled only collagen‐combination trials with identical complete‐closure constructs. Collagen‐alone was reported separately as a single‐trial estimate.

### 2.5 Study Quality Assessment Using the CASP Checklist [29]

The six studies (five randomized controlled trials and one singlge‐blinded non‐randomized) included in this review were systematically evaluated using CASP. This tool appraises trial quality across randomisation, blinding, baseline comparability, outcome measurement, statistical methods, and ethical reporting. A detailed narrative appraisal is provided below; a tabulated breakdown is available in Supplementary File S2. The complete dataset, including the two initially screened but later excluded RCTs (due to limited access/full‐text unavailability), is documented in Supplementary File S1, ensuring full transparency.

#### 2.5.1 Quality Appraisal (5 RCTs; 1 non‐randomized comparative study)

- Gould et al. (2022, International Wound Journal): Prospective, randomized, single‐blinded multicentre RCT (*n* = 100; six centres). Strengths included clearly defined eligibility, standardized wound measurement (eKare InSight), blinded outcome assessment, and ITT analysis. Significant improvements were seen in complete healing (74% vs 38%) and time‐to‐heal; neuropathy signals were also reported. Ethics approval and informed consent were stated [25].
- Djavid et al. (2020, Journal of Wound Care): Open‐label, single‐centre RCT (*n* = 61) comparing collagen‐chitosan with saline‐gauze. Healing favoured the intervention (60% vs 35.5%) with greater early area reduction (54.5% vs 38.8%). No significant device‐related adverse events were reported. Trial registration: IRCT20170612034485N1; Ethics: IR.ACECR.IBCRC.REC.1396.12 [31].
- Park et al. (2019, Diabetes Research and Clinical Practice): Prospective, randomized, placebo‐controlled, single‐centre RCT (*n* = 30). Strengths: well‐defined eligibility, blinded outcome assessment (Visitrak Digital Planimetry), and appropriate statistics (Statistical Analysis System [SAS]; Kaplan–Meier). Healing significantly higher with collagen (82.4% vs. 38.5%, *p* = 0.022) healing velocity improved, and time to 50% reduction was shorter (21 vs 42 days; HR 1.94, *p* <.05). Clinical staff performing DFU evaluations were unaware of group allocation; participant and treating‐clinician blinding was not claimed. Registration: Clinical Research Information Service (CRIS), identifier KCT0003802 [30].
- Gottrup et al. (2013, Wound Repair and Regeneration): Open‐label randomized trial (*n* = 390) comparing collagen–ORC/silver with standard care. Randomisation via sealed envelopes; no blinding reported. Outcomes measured with Visitrak, Quantify One and biochemical assays. Results showed superior 4‐week responder rate (≥ 50% area reduction: 79% vs. 43%, *p* = 0.035) and reduced infection‐related withdrawal (0% vs. 31%, *p* = 0.012). Closure at 14 weeks was also reported. Ethics: written informed consent was obtained before enrolement; the protocol was approved by the Regional Scientific Ethical Committee and the study was conducted in accordance with the Declaration of Helsinki and Good Clinical Practice [32].
- Kakagia et al. (2007, Journal of Diabetes and Its Complications): Three‐arm RCT (*n* = 51) comparing ORC/collagen, autologous growth factors delivered by the Gravitational Platelet Separation (GPS) system, and their combination, over 8 weeks in outpatients with DFUs. Randomization was performed with a random number generator; blinding not described. ImageTool planimetry was used, with analyses via SPSS (ANOVA/Tukey). The combination arm achieved the greatest ulcer‐size reduction; complete healing at 8 weeks was limited across arms. Ethics: all participants provided written informed consent; the study followed Democritus University (Alexandroupolis) clinical‐trial guidelines and the Declaration of Helsinki [33].
- Lobmann et al. (2006, Journal of Diabetes and Its Complications): Single‐blinded comparative study, (*n* = 33; Texas 2A DFUs). Emphasis on 8‐day biochemical endpoints (ELISA): decreased MMP‐9/TIMP‐2 ratio and greater early surface‐area reduction (16% vs 1.6%, *p* = 0.045); IL‐1β. University of Magdeburg ethics approval reported; single‐blinded design confirmed [22].

#### 2.5.2 Supplementary Materials

- Supplementary Material (S1–S5) for search strategies, CASP/GRADE, Summary of Findings,per trial details, and meta‐analysis notes.
- Calculations and data extraction are provided in Supplement S5.

### 2.6 Quality Appraisal Summary

Methodological quality was heterogeneous: the highest‐quality RCT was Park 2019 (randomized, assessor‐blinded, validated outcomes); Djavid 2020 and Kakagia 2007 were randomized but open‐label/with incomplete blinding; Gottrup 2013 was open‐label with limited randomization detail. Lobmann 2006 was single‐blinded, non‐randomized with an 8‐day biochemical focus and is reported narratively only (not eligible for pooled RCT analyses).

Gould 2022 was a single‐blind, multicentre RCT of PMVT vs collagen‐alginate and was not pooled due to its mechanistically distinct intervention.

### 2.7 Protocol Registration (PROSPERO)

**FIGURE 3:**
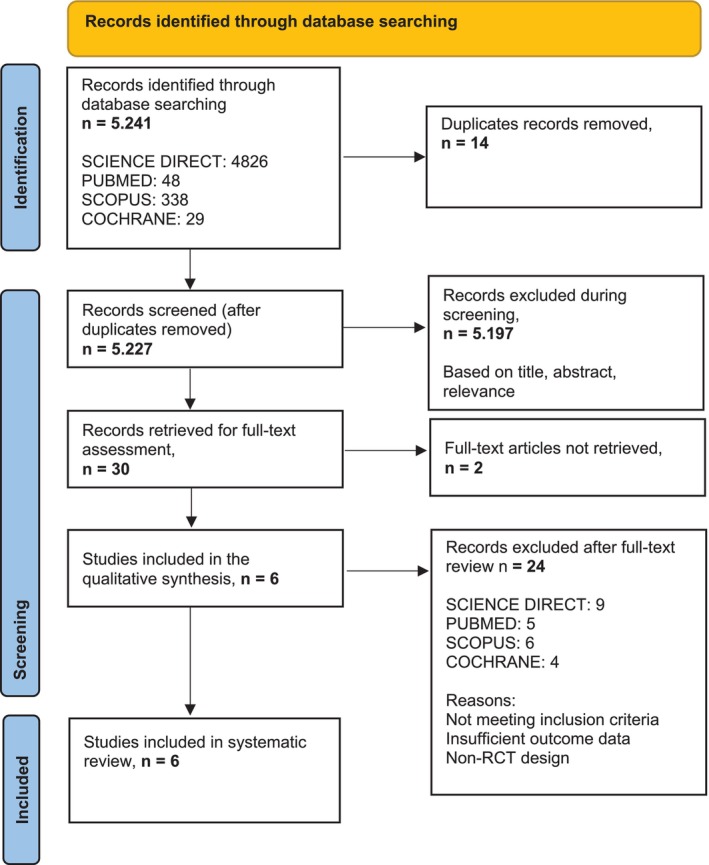
Preferred Reporting Items for Systematic Reviews and Meta‐Analyses (PRISMA) [34].

This review was not prospectively registered in PROSPERO. To mitigate risks of selective reporting or unplanned analytic choices, we (i) prespecified the PICO [26], eligibility criteria, databases, and filters (S1/S1b); (ii) provide full search strings, database counts, and a PRISMA32 flow (Figure 3); (iii) present study‐level CASP33 appraisals (S2/S4) and a transparent Summary of Findings (SoF)/GRADE summary (S3); and (iv) predefined a restricted meta‐analysis focused solely on complete closure (yes/no, ITT) in clinically comparable collagen‐combination RCTs. All quantitative calculations and the workbook (DFU_meta_2025.xlsx) are provided in S5. Future updates should be prospectively registered (e.g., PROSPERO/OSF) with any protocol deviations explicitly documented.

## 3 Results

### 3.1 Study Selection

The database search yielded 5241 records. After removal of duplicates and screening, six included studies (five RCTs and one single‐blinded non‐randomized) (total *n* = 314) were eligible (PRISMA flowchart, Figure 3).

### 3.2 Study Characteristics

The included five RCTs and one comparative study were published between 2006 and 2022, with sample sizes ranging from 30 to 100. Four were single‐centre trials; Gould et al. (2022) was multicentre. Follow‐up ranged from 4 to ~20 weeks depending on the prespecified endpoint (e.g., early ‘responder’ at 4 weeks; complete closure at 12–14 weeks; one study analysed up to ~24 weeks). Interventions included collagen alone or collagen combined with adjuncts (e.g., oxidised regenerated cellulose [ORC], silver, chitosan). Comparators ranged from saline gauze or foam (placebo) to standard advanced dressings/standard of care (SOC). A concise summary appears in Table 1, with full methodological details in Table S3.

**TABLE 1:** Characteristics and main findings of included studies (5 RCTS + 1 single‐blinded comparative study*).

Study (year)	Design and Sample	Intervention vs Comparator	Follow‐up/Key Endpoint(s)	Main Findings
Gould et al., 2022	Prospective, randomized, single‐blinded multicentre RCT; *n* = 100	PMVT allograft vs collagen‐alginate	12 weeks (closure)	Higher complete closure 74% vs 38%; faster time‐to‐heal; neuropathy signal.
Djavid et al., 2020	Open‐label, single‐centre RCT; *n* = 61	Collagen‐chitosan vs saline gauze + SOC	20 weeks (closure); early area change	Complete closure 60.0% vs 35.5%; area reduction 54.5% vs 38.8%.
Park et al., 2019	Prospective, randomized, placebo‐controlled, single‐centre RCT; *n* = 30	Porcine type I collagen vs foam (placebo) + SOC	12 weeks (closure); healing velocity; time to 50% reduction	Closure 82.4% vs 38.5% (*p* = 0.022); faster healing; 50% reduction 21 vs 42 days (HR 1.94, *p* < 0.05).
Gottrup et al., 2013	Open‐label randomized trial; *n* = 39	Collagen‐ORC + silver vs SOC	4 weeks (≥ 50% area responder); 14 weeks (closure)	Responders 79% vs 43% (*p* = 0.035); infection‐related withdrawals 0% vs 31% (*p* = 0.012); closure 12/23 vs 4/13.
Kakagia et al., 2007	Three‐arm RCT; *n* = 51 (17/17/17)	ORC/collagen; autologous growth factors (GPS); combination	8 weeks	Combination arm had greatest ulcer‐size reduction (*p* < 0.001); complete healing limited across arms.
Lobmann et al., 2006	Single‐blinded, non‐randomized comparative study; *n* = 33 (Texas 2A DFUs)	Collagen‐ORC vs SOC	4 and 8 day (biomarkers); short clinical follow‐up	↓ MMP‐9/TIMP‐2 (*p* < 0.05); ↑ IL‐1β (*p* = 0.01); surface reduction −16% vs. −1.6% (*p=*0.045).

*Note:* All pooled meta‐analysis calculations (study 2 × 2 tables; per‐study log(RR)/SE; pooled fixed & random models; *Q*, *p*, *I* ^2^, *τ* ^2^; 95% prediction interval) are documented in Supplement S5 and the Excel workbook DFU_meta_2025.xlsx.

Abbreviations: DFU, diabetic foot ulcer; IL‐1β, interleukin‐1 beta; ITT, intention‐to‐treat; MMP‐9, matrix metalloproteinase‐9; ORC, oxidised regenerated cellulose; PMVT, processed microvascular tissue allograft; SOC, standard of care; TIMP‐2, tissue inhibitor of metalloproteinases‐2.

### 3.3 Overall Patterns

Despite variation in design, comparators, and outcome definitions, a consistent trend favoured collagen‐based interventions—particularly collagen combinations (e.g., collagen–ORC/silver; collagen–chitosan)—for accelerating healing versus control. However, differences in ulcer classification systems, small samples, short follow‐up windows, and non‐standardised endpoints limit certainty and generalisability. These considerations informed our decision to conduct a restricted meta‐analysis only where outcome constructs were identical.

### 3.4 Focused Meta‐Analysis (Pre‐Specified)

We pooled complete wound closure (yes/no, intention‐to‐treat [ITT]) at each trial's final prespecified follow‐up for collagen‐combination dressings vs. control from two RCTs (Djavid et al. 2020; Gottrup et al. 2013). Using a Mantel–Haenszel fixed‐effect model (with DerSimonian–Laird random‐effects as sensitivity), the pooled effect was:

- RR 1.69 (95% CI 1.05–2.72), indicating a higher probability of complete closure with collagen‐combination dressings. Heterogeneity: *Q* = 0.00003, df = 1, *p* = 0.999987; *I* ^2^ = 0.0%; *τ* ^2^ = 0.00000. Random‐effects 95% prediction interval: 1.05–2.72.

Note: *k* = 2; one trial used saline gauze as a control and adjuncts differed (chitosan vs. ORC/silver). We therefore treat the pooled estimate as a direction‐of‐effect summary rather than a time‐standardised magnitude.

The meta‐analysis used the following ITT closure counts: Gottrup et al. 2013: 12/23 vs. 4/13; Djavid et al. 2020: 18/30 vs. 11/31. Because adjuncts (chitosan vs. ORC/silver) and follow‐up durations (~24 weeks vs. 14 weeks) differed, this pooled estimate is interpreted as a direction‐of‐effect summary rather than a time‐standardised magnitude. All pooled calculations (fixed and random models, *Q*, *p*, *I* ^2^, *τ* ^2^, prediction interval) are provided in Supplement S5 and the Excel workbook (DFU_meta_2025.xlsx).

## 4 Discussion

Collagen‐based dressings—particularly collagen–oxidised regenerated cellulose (ORC) and other collagen‐combination formulations—are biologically plausible therapies for chronic diabetic foot ulcers (DFUs). By binding excess proteases, preserving endogenous growth factors, stabilising the extracellular matrix (ECM), and modulating inflammation, they can promote granulation and re‐epithelialisation. Across the six studies included, these dressings were generally associated with faster healing, greater wound contraction, and—in some studies—lower infection‐related events compared with control.

### 4.1 What the Quantitative Synthesis Adds

To avoid inappropriate pooling, we meta‐analysed only complete wound closure (yes/no, intention‐to‐treat [ITT]) from trials using collagen‐combination dressings with comparable constructs at each study's final prespecified follow‐up. The pooled effect (two RCTs) suggested a higher probability of complete closure vs. control (RR 1.69, 95% CI 1.05–2.72). Heterogeneity: *Q* = 0.00003, df = 1, *p* = 0.999987; *I* ^2^ = 0.0%; *τ* ^2^ = 0.00000. Random‐effects 95% prediction interval: 1.05–2.72. Because adjuncts (chitosan vs. ORC/silver) and follow‐up windows (~20 vs. 14 weeks) differed, this estimate is best interpreted as a direction‐of‐effect summary rather than a time‐standardised magnitude. In parallel, a single blinded RCT of collagen‐alone reported superior 12‐week closure versus a placebo dressing, supporting the overall signal while remaining non‐poolable. Biomarker improvements (e.g., reduced MMP‐9/TIMP‐2) and early responder outcomes provide mechanistic and short‐term clinical support for this signal. All pooled calculations (fixed and random models, *Q*, *p*, *I* ^2^, *τ* ^2^, and prediction interval) are documented in Supplement S5 and the Excel workbook (DFU_meta_2025.xlsx).

### 4.2 Limitations of the Evidence Base

Several features temper certainty: (i) small, mostly single‐centre RCTs (*n* often < 100); (ii) heterogeneous comparators (from saline gauze or foam to advanced dressings with silver), complicating isolation of collagen–ORC's independent effect; (iii) non‐standardised endpoints and timepoints (complete closure vs. ≥ 50% area reduction vs. biomarkers), with short follow‐up (often 4–12 weeks) that do not address durability, recurrence, or limb outcomes; and (iv) incomplete control/reporting of confounders (infection treatment, vascular status/revascularisation, off‐loading modality/adherence, and glycaemic control). Differences in ulcer classification and measurement tools (eKare InSight, AutoCAD, Visitrak; Wagner vs. Texas 2A) further constrain cross‐trial comparability [27, 28]. One pooled RCT used saline gauze as the control, which is non‐standard in contemporary DFU care, further tempering certainty.

### 4.3 Risk of Bias and Sponsorship

Several trials were open‐label with limited allocation detail, increasing performance/detection bias risk. Industry sponsorship was present in some studies (e.g., collagen/ORC/silver and processed microvascular tissue [PMVT]), underscoring the need for independent, investigator‐led confirmatory RCTs. These factors contributed to moderate certainty for closure with collagen‐combination dressings (GRADE) and moderate certainty for collagen‐alone based on a single RCT, with low certainty for early responder/biomarker outcomes.

### 4.4 Protocol Registration (PROSPERO) and Reporting Transparency

This review was not pre‐registered in PROSPERO, introducing the potential risk of selective reporting or unplanned analytical decisions. We mitigated this by prespecifying PICO [26], eligibility, databases/filters (S1), publishing the full search strings and PRISMA [34] flow (S1/S1b; Figure 3), providing study‐level CASP appraisals (S2/S4) and a transparent Summary of Findings (SoF)/GRADE table (S3), and restricting the meta‐analysis to a single, predefined outcome (complete closure, yes/no, ITT) in clinically comparable collagen‐combination RCTs, with a calculations workbook in Supplement S5 (DFU_meta_2025.xlsx). Even so, future reviews should be prospectively registered (e.g., PROSPERO/OSF) with any deviations explicitly documented.

### 4.5 Clinical Implications

Within a comprehensive DFU pathway—timely debridement, infection control, strict off‐loading, vascular assessment/intervention, and glycaemic optimisation—collagen‐based dressings appear to increase the likelihood of closure over the comparators used in the included trials. Given the short horizons and heterogeneous controls, these products should be viewed as adjuncts to gold‐standard care rather than stand‐alone solutions, with product selection tailored to wound phenotype (e.g., highly inflammatory/exudative ulcers likely to benefit from protease modulation).

### 4.6 Research Priorities

Future trials should (i) pre‐register protocols with harmonised closure definitions and ≥ 24–52‐week follow‐up to assess durability/recurrence and limb endpoints; (ii) standardise and report off‐loading, antimicrobial management, and vascular status to minimise confounding; (iii) include time‐to‐heal, amputation, and health‐economic outcomes; and (iv) compare collagen‐alone vs. specific collagen‐combinations head‐to‐head to define incremental benefit. Stratified designs by neuropathic vs. neuro‐ischaemic DFUs and by baseline protease activity may clarify which patients benefit most.

### 4.7 In conclusion

Collagen‐based dressings—especially collagen‐combination formulations—likely improve complete closure in DFUs when added to standard care. The effect is promising but based on small, short‐term RCTs with methodological heterogeneity; recommendations should be measured pending larger, longer, and more standardised trials. Numerically, the pooled estimate is RR 1.69 (95% CI 1.05–2.72; *Q* ≈ 0, *I* ^2^ = 0%, *τ* ^2^ = 0; random‐effects 95% PI ≈ 1.05–2.72), with full calculations available in Supplement S5 and DFU_meta_2025.xlsx.

## 5 Conclusion

Collagen‐based dressings—particularly collagen–oxidised regenerated cellulose (ORC) and other collagen‐combination formulations—show promising adjunctive benefit in diabetic foot ulcer (DFU) care. Signals across the six included studies five RCTs and one single‐blinded non‐randomized include higher rates of complete closure, greater early wound reduction, and favourable protease modulation. In a restricted meta‐analysis pooling only comparable trials and outcomes (complete closure, yes/no, intention‐to‐treat [ITT]; collagen‐combination vs. control), the probability of closure was higher with collagen‐combination dressings (risk ratio 1.69, 95% confidence interval 1.05–2.72; *I* ^2^ = 0%), supporting a beneficial direction of effect. Heterogeneity was negligible (*Q* = 0.00003, df = 1, *p* = 0.999987; *τ* ^2^ = 0.00000), and the random‐effects 95% prediction interval was 1.05–2.72. Full pooled calculations are provided in Supplement S5 and the Excel workbook (DFU_meta_2025.xlsx).

However, certainty remains limited by small, mostly single‐centre samples; heterogeneous comparators; non‐standardised outcomes and follow‐up windows; incomplete control/reporting of confounders (infection management, vascular status, off‐loading, glycaemic control); open‐label designs; and industry sponsorship in some trials. These constraints preclude strong practice‐changing recommendations and warrant measured clinical adoption—positioning collagen‐based dressings as adjuncts within a comprehensive DFU pathway rather than stand‐alone solutions.

Future research should prioritise larger, multicentre RCTs with prospective registration, harmonised closure definitions, greater week follow‐up (durability/recurrence and limb outcomes), transparent reporting of off‐loading/vascular/antimicrobial care, and health‐economic analyses. Head‐to‐head comparisons of collagen‐alone versus specific collagen‐combinations are needed to define incremental benefit and identify which patients benefit most. Pending such data, collagen‐based dressings can be considered targeted adjuncts for appropriately selected DFUs within evidence‐based multidisciplinary care.

## Conflicts of Interest

The authors declare no conflicts of interest.

## Supporting information


**Data S1:** Supporting Information.

**Data S2:** Supporting Information.

## Data Availability

All data supporting the findings are available in the article and its Supplementary Materials.
