# Supplementary material for: Collagen–ORC Versus Standard Treatment in Diabetic Foot Ulcers: A Systematic Review and Meta‐Analysis of Randomised Trials
Source: Int Wound J. 2025 Nov 17;22(11):e70782. doi: 10.1111/iwj.70782 (PMC12623144; doi:10.1111/iwj.70782)
Supplement: Supplementary file 1 — Data S1: Supporting Information. [file IWJ-22-e70782-s002.docx]

**Supplementary Material — Collagen-ORC Versus Standard Treatment in Diabetic Foot Ulcers**

# **S1. Detailed Search Strategy**

Framework: We used a structured PICO to evaluate collagen‑based dressings for diabetic foot ulcers (DFUs).

| **PICO element** | **Definition** |
| --- | --- |
| Population | Adults with DFUs (neuropathic and/or ischemic). |
| Intervention | Collagen‑based dressings (collagen alone; collagen–oxidized regenerated cellulose [collagen–ORC]; combinations, e.g., collagen–chitosan or collagen/ORC/silver). |
| Comparator | Standard of care (SOC) or alternative standard dressings. |
| Outcome | Wound healing (complete closure as the primary pooled endpoint). |

Databases & dates: PubMed, ScienceDirect, Scopus, and Cochrane Library; searches performed March 2025.

Filters (applied as available): randomized controlled trials, English language, publication years 2005–2025.

## **S1.1 Search algorithms by database**

PubMed (Mar 2025): Boolean combinations of “diabetic foot ulcers”, “standard of care”, and (“collagen dressing” OR “oxidized regenerated cellulose” OR “collagen matrix”), with RCT/English/2005–2025 filters. Initial ≈48 records; screened ≈17; included after deduplication: 2–4 relevant, with duplicates removed.

ScienceDirect (Mar 2025): Initial ≈4,826; filtered to full‑length research in Medicine/Nursing; ≈279 screened; ≈3 relevant.

Scopus (Mar 2025): Initial ≈338; after filters ≈126; ≈2 relevant unique after removing duplicates.

Cochrane Library (Mar 2025): ≈29 initial; after filters ≈25; all relevant items were duplicates captured elsewhere; no additional unique trials included.

# **S1b. Study identification and quality appraisal (CASP)**

Five RCTs and one non‑randomized mechanistic study met eligibility and were appraised with CASP. Narrative judgements are summarized in S2 and S4; key per‑study notes follow.

## Trial‑level CASP snapshots

- Djavid et al., 2020 (collagen–chitosan vs gauze + SOC): open‑label; groups comparable; ITT used; higher closure with collagen–chitosan (~60% vs ~36% at ~24 weeks).
- Gould et al., 2022 (PMVT allograft vs collagen–alginate): single‑blind assessors; 74% vs 38% closure at 12 weeks; mechanistically different from collagen and not pooled.
- Lobmann et al., 2006 (NON‑randomized mechanistic study; collagen–ORC vs SOC): assessor‑blinded biomarker assessment; short horizon (4–8 days); ↓MMP‑9/TIMP‑2; not pooled for closure.
- Kakagia et al., 2007 (collagen, autologous growth factors, combination): open‑label three‑arm; combination best (P<.001); extractable closure counts not reported.
- Park et al., 2019 (collagen vs foam + SOC): assessor‑blinded outcomes; 12‑week closure 82.4% vs 38.5% (RR 2.14, 95% CI 1.09–4.22).
- Gottrup et al., 2013 (collagen/ORC/silver vs SOC): open‑label; 4‑week responder advantage and higher 14‑week closure (RR ≈1.70); fewer infection‑related withdrawals (0% vs 31%).

# **S2. CASP‑based Risk of Bias Appraisal and GRADE Evidence Profile**

## S2.1 Risk of Bias (CASP summary)

| **Study** | **Design** | **Randomization / Concealment** | **Blinding** | **Baseline Comparability** | **Outcome Measurement** | **Attrition / ITT** | **Overall RoB** |
| --- | --- | --- | --- | --- | --- | --- | --- |
| Gould 2022 | RCT | Randomized; concealment reported | Assessor‑blind | Comparable | Validated closure metrics | Adequate; ITT | Some concerns |
| Djavid 2020 | RCT | Randomized; concealment unclear | Open‑label | Comparable | Complete closure; planimetry | Adequate; ITT ≈100% | High |
| Park 2019 | RCT | Adequate sequence; concealment unclear | Assessor‑blind | Comparable | Visitrak®; predefined ITT | Low attrition | Some concerns |
| Gottrup 2013 | RCT | Randomized; method NR | Open‑label | Balanced | Objective clinical endpoints | Adequate; withdrawals described | High |
| Kakagia 2007 | RCT (three‑arm) | Randomized; sequence unclear | Open‑label | Comparable | Size/healing reduction | Adequate | High |
| Lobmann 2006 | Non‑randomized mechanistic study | N/A | Assessor‑blinded biomarker assessment | Comparable | Biomarkers + area | Short horizon | Some concerns |

## **S2.2 GRADE evidence profile — key outcomes**

Question: In adults with DFUs, do collagen‑based dressings improve healing compared with standard dressings/SOC? Only RCTs were included in meta‑analysis and GRADE for the closure outcome; Lobmann 2006 (non‑randomized mechanistic) informs mechanism only.

| **Outcome** | **Comparison / Studies (k)** | **Effect** | **Certainty (GRADE)** | **Key reasons** |
| --- | --- | --- | --- | --- |
| Complete closure (final follow‑up, ITT) | Collagen‑combination vs control; k=2 (Djavid 2020; Gottrup 2013) | RR 1.69 (95% CI 1.05–2.72); I²≈0% | MODERATE | Some RoB; consistency acceptable; imprecision limited. |
| Complete closure (12 w, ITT) | Collagen‑alone vs control; k=1 (Park 2019) | RR 2.14 (95% CI 1.09–4.22) | MODERATE | Single small RCT; downgraded for imprecision. |
| Responder ≥50% area reduction (4 w) | Collagen‑combination vs control; k=1 (Gottrup 2013) | RR ~1.85 (CI wide) | LOW | Single small RCT; imprecision. |
| Safety (AEs/infection‑related) | Mixed; mainly Gottrup 2013 | No consistent device‑related harms; fewer infection‑withdrawals with collagen/ORC/silver | LOW | Sparse AE reporting; small samples. |

# **S3. Summary of Findings (SoF) and Methods Notes**

| **Intervention** | **Outcome (timepoint)** | **Effect** | **Absolute effect (illustrative)** | **Certainty** |
| --- | --- | --- | --- | --- |
| Collagen‑combination (Djavid 2020 + Gottrup 2013) | Complete closure (final follow‑up) | RR 1.69 (95% CI 1.05–2.72); I²≈0% | If 350/1000 heal with control → ~592/1000 with collagen‑combination (range ~368–952/1000) | MODERATE |
| Collagen‑alone (Park 2019) | Complete closure (12 w) | RR 2.14 (95% CI 1.09–4.22) | If 385/1000 heal with control → ~825/1000 with collagen (range ~420–1000/1000) | MODERATE |
| Collagen‑combination | Responder ≥50% area↓ (4 w) | RR ~1.85 (CI wide) | Direction favors collagen; absolute change uncertain | LOW |
| Collagen‑combination | Adverse events | No consistent device‑related harms; fewer infection‑withdrawals in one RCT | — | LOW |

## Statistical approach (focused and conservative)

We pooled only complete closure (yes/no, ITT) where ≥2 trials shared compatible definitions/timepoints (Djavid 2020; Gottrup 2013).

Mantel–Haenszel fixed‑effect risk ratios were primary; DerSimonian–Laird random‑effects used as sensitivity; heterogeneity described but not over‑interpreted with k=2.

Other endpoints (4‑week responder, time‑to‑heal, biomarkers) were not pooled due to construct/timepoint incompatibilities.

Lobmann 2006 is explicitly treated as a non‑randomized mechanistic study (biomarkers/short horizon) and is not counted among RCTs nor included in pooled estimates.

# **S4. Detailed outcomes and risk of bias by trial**

| **Study (Year)** | **Design / Setting** | **Sample (INT/CTRL/Total)** | **Intervention vs Comparator** | **Follow‑up** | **Primary endpoint** | **Key results** | **Adverse events / Notes** |
| --- | --- | --- | --- | --- | --- | --- | --- |
| Park 2019 | RCT; assessor‑blinded; single‑center | 17/13/30 | Collagen vs foam + SOC | 12 w | Complete closure (Visitrak®), ITT | 14/17 vs 5/13 → RR 2.14 (95% CI 1.09–4.22) | No device‑related SAEs |
| Djavid 2020 | RCT; open‑label; single‑center | 30/31/61 | Collagen–chitosan vs gauze + SOC | ~24 w | Complete closure (planimetry), ITT | 18/30 vs 11/31 → RR ≈1.69 | No significant device‑related AEs |
| Gottrup 2013 | RCT; open‑label; multicenter | 24/15/39 (4 w); 23/13 (14 w) | Collagen/ORC/silver vs SOC | 4 w; 14 w | Responder ≥50% area (4 w); closure (14 w) | 4 w: 19/24 vs 6/14 (p=0.035); 14 w closure: 12/23 vs 4/13 (RR ≈1.70) | Fewer infection‑related withdrawals (0% vs 31%) |
| Kakagia 2007 | RCT; three‑arm; open‑label | 17/17/51 | Collagen; autologous GF; combination | ≤20 w | Ulcer reduction & healing | Combination best (P<.001); closure counts NR | Quantitative closure not extractable |
| Lobmann 2006 | Non‑randomized mechanistic study; assessor‑blinded biomarkers | 16/17/33 | Collagen–ORC vs SOC | 4 and 8 d | MMP‑9/TIMP‑2; area | ↓MMP‑9/TIMP‑2; area −16% vs −1.6% | Mechanistic; short horizon; not pooled for closure |
| Gould 2022 | RCT; single‑blind; multicenter | — / — / 100 | PMVT allograft vs collagen–alginate | 12 w | Complete closure (eKare®), ITT | 74% vs 38% (context only; not pooled) | Mechanistically distinct from collagen |

Abbreviations: SOC, standard of care; ITT, intention‑to‑treat; ORC, oxidized regenerated cellulose; Ag, silver; GF, growth factors; NR, not reported.

# **S5. Meta‑analysis workbook (text summary)**

Aligned RCTs pooled: Djavid 2020 and Gottrup 2013 — both report complete wound closure (yes/no) with compatible denominators under ITT at each study’s final follow‑up (≈24 w and 14 w, respectively).

| **Study** | **Events (T)** | **Non‑events (T)** | **Events (C)** | **Non‑events (C)** | **Risk_T** | **Risk_C** | **RR (95% CI)** |
| --- | --- | --- | --- | --- | --- | --- | --- |
| Gottrup 2013 | 12 | 11 | 4 | 9 | 0.522 | 0.308 | 1.70 (0.69–4.19) |
| Djavid 2020 | 18 | 12 | 11 | 20 | 0.600 | 0.355 | 1.69 (0.97–2.95) |

| **Pooled model** | **RR (95% CI)** | **k** | **Q (df)** | **p(Q)** | **I²** | **τ² (DL)** | **Prediction interval** |
| --- | --- | --- | --- | --- | --- | --- | --- |
| Fixed‑effect (primary) | 1.69 (1.05–2.72) | 2 | 0.00003 (1) | 0.999987 | 0.0% | 0.00000 | 1.05–2.72 |
| Random‑effects (DL) | 1.69 (1.05–2.72) | 2 | — | — | — | — | — |

Interpretation: Collagen‑combination dressings improved the probability of complete closure vs control at final follow‑up (moderate certainty). With k=2, between‑study heterogeneity estimates are unstable and not over‑interpreted.

Clarification: Lobmann 2006 is a non‑randomized mechanistic study and is not treated as an RCT anywhere in this corrected file.

**S5a. Meta-analysis workbook and independent verification**
The complete meta-analysis for healing outcomes was conducted in Microsoft Excel (DFU_meta_2025.xlsx), which contains the extracted 2×2 data, study-level risk ratios, pooled estimates, and heterogeneity statistics.

For transparency and reproducibility, the primary analysis was independently re-run in MetaAnalysisOnline using identical inputs and model choices (Mantel–Haenszel fixed effect and DerSimonian–Laird random effects). The resulting forest plots confirm the reported pooled risk ratio and low between-study heterogeneity. These verification plots are shown below as part of Supplement S5 (Figures S5A–S5B).

**Figure S5A. Forest plot from MetaAnalysisOnline (fixed-effect Mantel–Haenszel model) confirming the pooled effect of collagen–ORC versus standard treatment on complete closure in diabetic foot ulcers.**

**Figure S5B. Forest plot from MetaAnalysisOnline (random-effects model) confirming the robustness of the pooled effect and low heterogeneity.**
